# Strengths-Based Interprofessional Practice and Education: Transforming Care Through Disruption

**DOI:** 10.1093/geroni/igab046.2203

**Published:** 2021-12-17

**Authors:** Teri Kennedy

**Affiliations:** The University of Kansas Medical Center, Kansas City, Kansas, United States

## Abstract

This is a conceptual paper proposing a new model of Strengths-Based Interprofessional Practice and Education (SB-IPE), incorporating appreciative inquiry and narrative, and its application to improve health and social care practice and policy for older adults. Within people, families, communities, and teams are people who understand their assets and culture, hold a collective wisdom derived from their individual biographies and shared history, and are deeply invested in their success. This wisdom and experience can be mined for strengths and best practices to improve health and social care for older adults and their families. The conceptual framework of the model and relationship between concepts are explained, reviewing and synthesizing relevant literature on the strengths perspective, interprofessional practice and education, evolution of the patient voice, appreciative inquiry, and narrative to leverage the voices and experiences of older adults, their families, and interprofessional teams. Providing person-, family-, and community-centered health and social care through SB-IPE involves eliciting, listening to, and processing stories and narratives, then coalescing and co-creating person/family/team narratives throughout the trajectory of care. Appreciate inquiry and narrative can be harnessed to imagine an improved experience of care for older adults and their families. Incorporating the potential disruption of the voices and perspectives of older adults and their families offers value for health and social care delivery and policy innovation. Application of the SB-IPE model holds promise for harnessing these voices and collective experiences leading from disruption to transformation of health and social care practice, health professions education, policy, and research.

